# Shielding the Pier Abutment Using a Nonrigid Connector

**DOI:** 10.7759/cureus.52895

**Published:** 2024-01-25

**Authors:** Ekta M Kanojia, Anjali Bhoyar, Surekha R Dubey, Seema Sathe, Sheetal R Khubchandani, Rakhi Shinde

**Affiliations:** 1 Prosthodontics, Sharad Pawar Dental College and Hospital, Datta Meghe Institute of Higher Education and Research, Wardha, IND

**Keywords:** nonrigid connector, stress breakers, key and keyway, semi-precision attachment, pier abutment

## Abstract

The attachment of a fixed partial denture (FPD) on a pier abutment may be necessary in some partially edentulous instances because of the pattern of lost teeth. Nonetheless, it has been noted that using a stiff FPD to restore two lost teeth and an intermediate pier abutment is not the best course of action. In this case, using a stiff connector concentrates the stresses on the pier abutment. In this situation, the pier abutment serves as a pivot, increasing the debonding of the fixed dental prosthesis and ultimately compromising the success of the FPD. Connectors that are not stiff can solve these issues. A nonrigid connector allows abutments to move independently and distributes shear forces to the supporting bone. Instead of the typical rigid connector, the nonrigid connector serves as a stress breaker between the retainer and the pontic.

## Introduction

A pier abutment or intermediate abutment is defined as a "natural tooth or implant abutment that is located between terminal abutments that serve to support a fixed or removable dental prosthesis" [1]. The retainers, pontic, and connectors are essential in transferring occlusal forces to the supporting tissue structures in a fixed partial denture (FPD). The FPD pontic firmly adheres to the retainer, providing stability and strength while reducing strains on the restoration [2]. The FPD longevity and its abutments depend on critical parameters such as the suitable length of the span, occlusion, abutment-related bone loss, and periodontal health. Overly flexing a long edentulous span FPD has an unfavorable prosthesis and material failure [3]. However, when a tooth has edentulous spaces on both sides, forming a pier abutment, a five-unit FPD may not be the optimal treatment because of physiologic tooth movement, abutment arch position, and variations in retainer retentive ability [4].

The treatment choice in restoring pier abutment cases is a dental implant or fixed movable bridge with a nonrigid connector using a precision or semi-precision attachment [5]. A fixed movable bridge with a nonrigid connector will serve as a stress breaker between the retainer and pontic, preventing stress transmission from the force applied to another prosthesis segment [6]. Various methods, including welding, soldering, and casting, are employed to create rigid connectors, and it is crucial to shape the cast connectors using wax patterns accurately. Nonrigid connectors allow for restricted movement between otherwise independent parts of FPDs. Creating nonrigid connectors can involve a custom milling machine, prefabricated plastic patterns, or the integration of prefabricated inserts [7]. This case study presents a straightforward rehabilitation technique for a pier abutment case using a prefabricated semi-precision attachment.

## Case presentation

**Figure 1:**
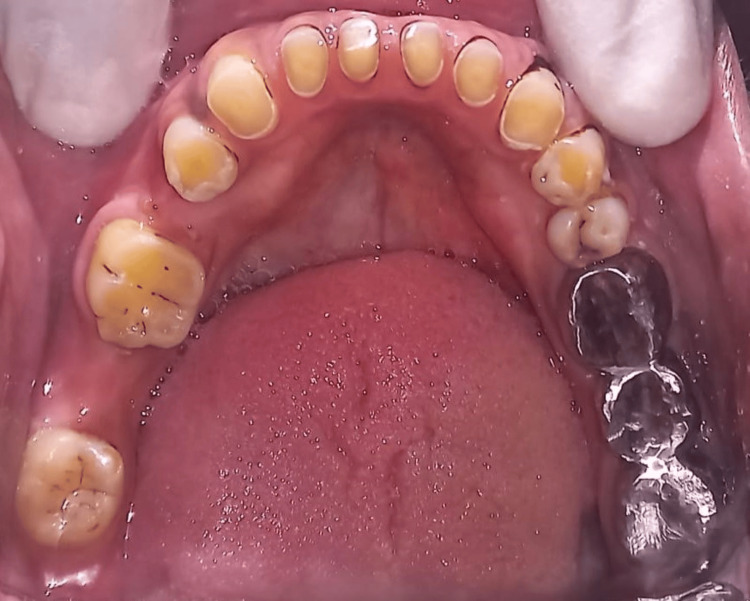
Image depicting the pier abutment.

A male 40-year-old patient had lost teeth in the right lower posterior region of the jaw for 10 months, and this was his main complaint when he came to the Department of Prosthodontics. Additionally, he reported having trouble with his teeth and having aesthetic issues. The patient's prior dental history indicated that the very carious teeth in their mandibular right second premolar and second molar had been extracted three months prior, but there is no pertinent medical history to report. Upon intraoral inspection, we discovered that the first molar was serving as a pier abutment and that the mandibular right second premolar and second molar were absent (Figure 1).

**Figure 2:**
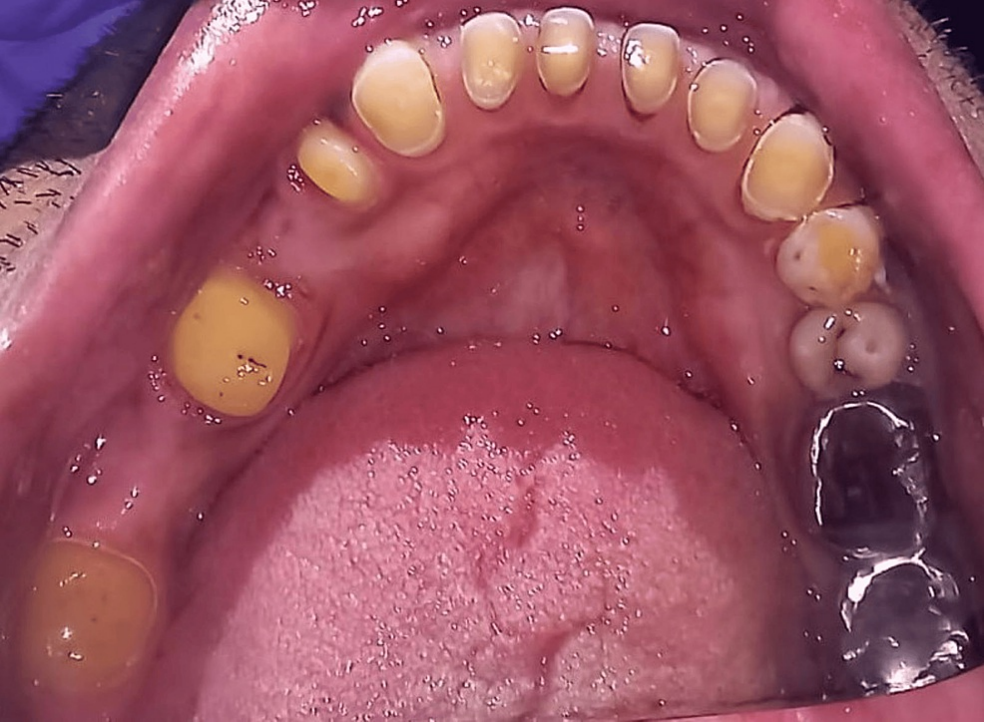
Tooth preparation of mandibular first premolar, first molar, and third molar.

Various treatment options were given to the patient, such as removable partial dentures and implant placement in the edentulous region. As the patient wanted a fixed partial prosthesis without any surgical intervention, both treatment options were ruled out, and a pier abutment with a nonrigid connector was selected as the treatment of choice. The oral rehabilitation process involved the following clinical actions. To improve the prosthesis outcome, tooth preparation was therefore completed on the mandibular right first premolar, first molar, and third molar, as shown in Figure 2, incorporating the equigingival borders and shoulder type of finish line.

A gingival retraction cord was used for the gingival retraction, and elastomeric impression material was used for the final impression, along with a two-stage putty wash procedure. To establish an accurate occlusion for the patient, an interocclusal record was created using bite registration material.

Provisional restorations were created using an auto-polymerizing acrylic resin in tooth color, and they were affixed with temporary cement that does not contain eugenol. A FPD, incorporating a nonrigid connector, was prepared. Initially, wax patterns were crafted for teeth 44, 45, and 46. On the distal surface of the wax pattern for tooth 46, a plastic castable male semi-precision attachment (Preci Vertix Male, Ceka Attachment, Belgium) was added during the waxing process. Surveying was undertaken to ascertain the position and parallelism of the plastic male attachment. Subsequently, investing and casting procedures were performed.

**Figure 3:**
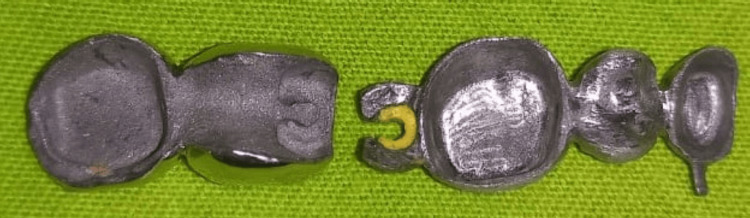
Metal coping with semi-precision attachments at 46 and 47.

The wax patterns for teeth 47 and 48 were crafted. A yellow elastic female component (Preci-Vertix Female, Ceka Attachment, Belgium) was positioned onto the previously casted male attachment. A recess for the male attachment was accurately carved to accommodate the elastic female on the mesial side of the wax pattern for tooth 47. The wax patterns for teeth 47 and 48 were meticulously removed after this step. Following the removal of the elastic female, the wax patterns for teeth 47 and 48 were invested and cast. The yellow elastic female was securely attached inside the casting at the specified recess, as illustrated in Figure 3.

**Figure 4:**
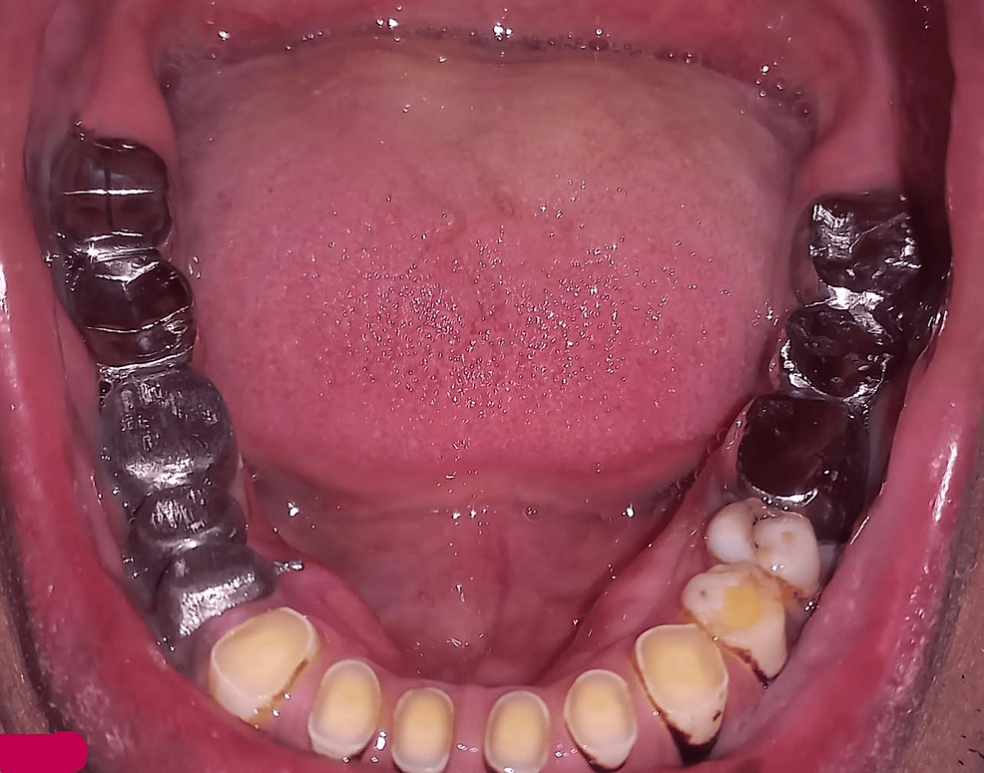
Metal try-in in the patient.

After casting, the metal try-ins of the individual units were done to verify the proper seating, as shown in Figure 4.

**Figure 5:**
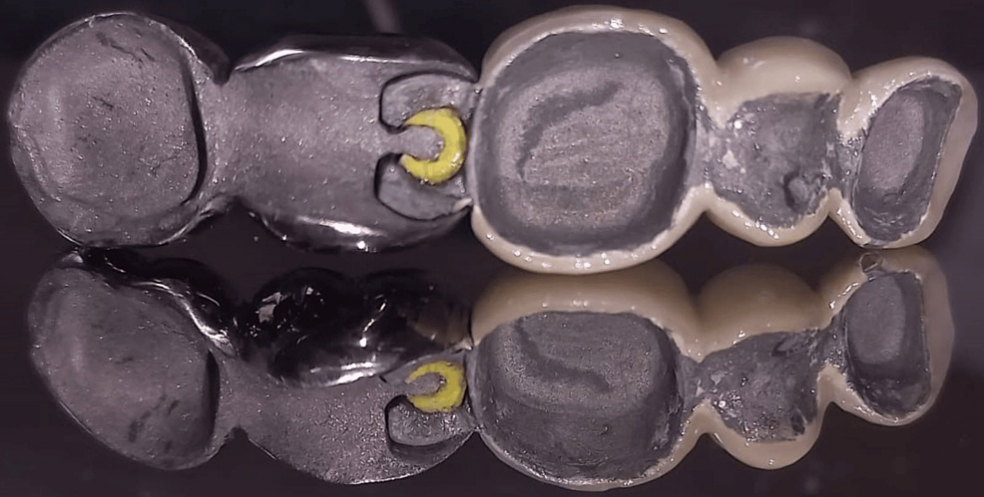
Intaglio completed five-unit fixed partial denture (FPD).

**Figure 6:**
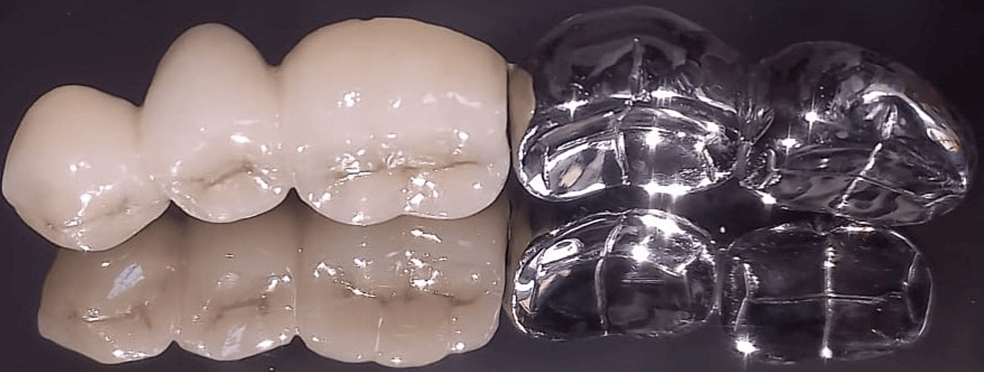
Completed five-unit FPD.

Next, a ceramic facing was incorporated into the structure. Moreover, the completed five-unit FPD is shown in Figures 5-6.

**Figure 7:**
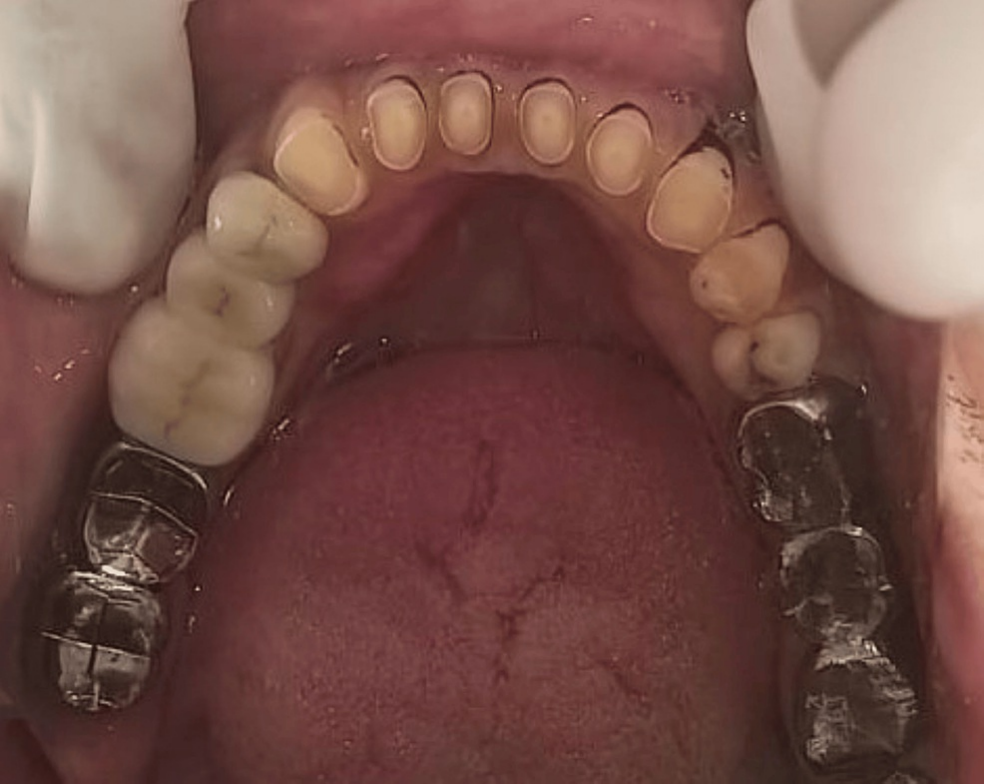
Finished cementation of a five-unit FPD.

During the cementation process, the mesial segment was affixed first, followed by the cementation of the distal segment. Type II glass ionomer cement (GC Fuji) was utilized for the cementation process, as depicted in Figure 7.

**Figure 8:**
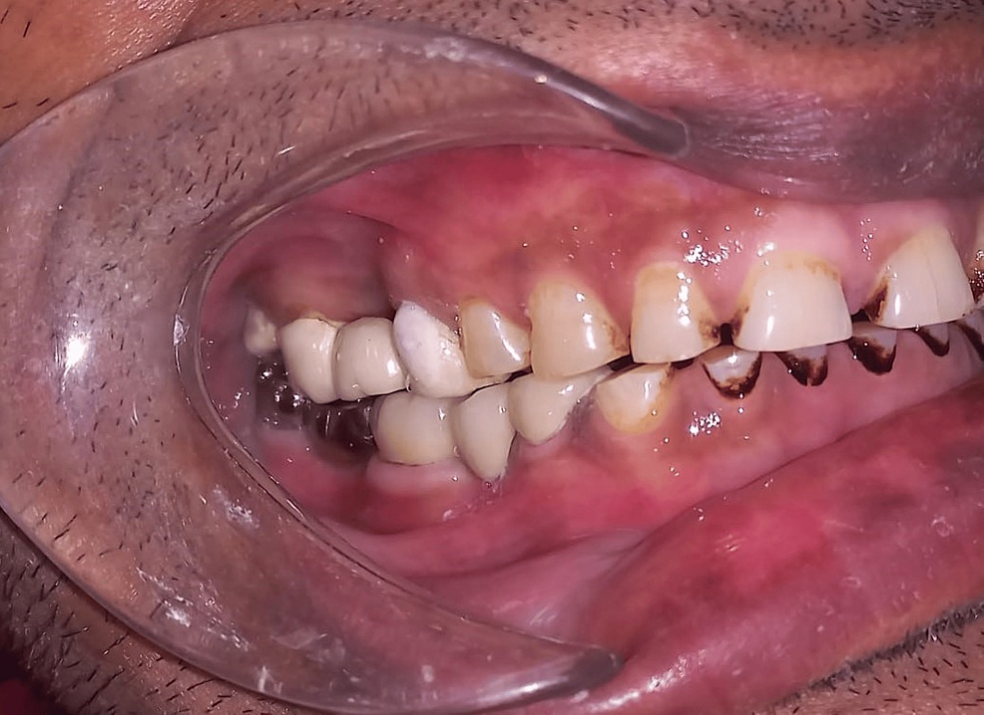
Post-clinical photograph of a five-unit FPD in occlusion.

The post-clinical photograph of a five-unit FPD in occlusion (Figure 8). The upper teeth also have crown restorations. The restoration material between the upper metal crown, which is porcelain tooth-colored material, and the lower metal crown is different, and this may cause attrition. 

The patient received instructions to uphold proper oral hygiene practices, with a specific recommendation to use dental floss and an interdental brush.

## Discussion

When there is a pier abutment present, nonrigid connectors should be used. When there is a misaligned abutment, on the other hand, intracoronal attachments should be used as connectors. In long-span prostheses, where porcelain may distort and shrink and result in an ill-fitting prosthetic, it is also utilized. Another crucial sign is when a patient has movable teeth and needs splinting as a treatment option. In these circumstances, interlocks are employed to stabilize the individual units. When abutment teeth exhibit significant mobility and splinting is not likely to result in a favorable outcome, nonrigid connections should not be used [8]. When more than one tooth is involved in the edentulous span, it is also contraindicated. In situations where there are inconsistencies and interferences concerning occlusal pressures and occlusion, nonrigid connectors were also not recommended.

In the case of a pier abutment, it is recommended to incorporate stress breakers at both ends of the nonrigid connector. This precaution is taken because shear pressures are directed toward the supporting bone rather than the connection in a stress breaker. By utilizing a stress breaker, abutments can move freely, thereby reducing mesiodistal torquing [9]. The presence of a pier abutment can create a fulcrum-like situation, potentially resulting in the intrusion of the pier abutment or the failure of the weakest terminal abutment [10].

Before the development of implant therapy, FPDs were the norm for treatment options. According to reports, the survival rate of fixed dental prostheses is 87% at 10 years and 69% at 15 years [11]. Nonvital anterior abutments and pier abutments were identified as critical variables contributing to failures [12]. Therefore, if there is adequate bone support and the patient can afford it, a dental implant may be a superior option in certain situations. Because the stiff and nonrigid connectors distribute less load on the abutments in the five-unit FPD, they can lengthen their lifespan. Moreover, it removes all obstacles to a fixed restoration with all stiff connectors and permits physiologic tooth movement [13]. According to Gill (1952), each tooth needs to be able to function both on its own and as a component of a larger whole, meaning that every tooth is essential to the mouth's overall health [14].

Using the finite element approach, Ziada et al. conducted a study and found that the distal part of the pier abutment had the least amount of stress concentration, supporting the nonrigid connectors placement in the distal region of the premolar [15]. While nonrigid connectors reduce shear stresses and torquing forces, they have a drawback. Clinically, more tooth reduction is required to facilitate engagement between male and female components, demanding higher accuracy and technique sensitivity. Reports also indicate instances of keys being lifted off when there is no occlusal stability [16]. Moreover, the distal pontic retainer and matrix to accommodate the matrix must be prepared, with the keyway in the distal part of the pier abutment and the key positioned mesially to the distalmost pontic. This arrangement ensures that mesial movements seat the key into the connector keyway if positioned on the distal side of the pier abutment [17].

Nonrigid connectors are of four types that have been identified in the literature: wing type and cross-pin, loop type, split type, and mortise-and-tenon type. Among these, the mortise-and-tenon type is the most popular, requiring precise mortise positioning to ensure parallelism for an accurate distal retainer withdrawal path. The success or failure of the restoration is contingent on the connector type used [18].

Unlike stiff FPD construction, which prevents independent abutment responses to vertical loading, the nonrigid design of FPD allows little independence in abutment reactions to vertical loads [19]. Quasi-3D photoelastic stress analysis studies have shown that continuous loading of FPDs leads to occlusal displacement and a certain level of stress [20].

Additionally, when a stress breaker is not recommended, one treatment option for a natural pier abutment is the use of two implants. However, thorough medical, clinical, and radiological examinations are necessary before implants can be inserted. Nonrigid connectors are recommended in situations where compromised conditions prevent the placement of implants. Therefore, precision and semi-precision attachments in five-unit FPDs contribute to an increased lifespan by allowing for minor adjustments that reduce the loading on the pier abutment because of its fulcrum-like condition [21].

## Conclusions

In cases requiring pier abutments, especially in five-unit bridges, rigid connectors are less desirable because of physiological teeth movement, abutment location to the arch, and the retainers' ability to hold teeth in place. The stiff attachment to two or more teeth in rigid connectors acts as "safety valves" against the enormous leverage pressures they create. The size, form, and type of connectors significantly influence the future success of an FPD. Nonrigid connectors, by reducing stress on the abutments, prolong the life of the restoration. The position of the nonrigid connector affects the stress distributions and values of the pier abutment and the FPD. Despite the longer turnaround times and higher laboratory costs associated with nonrigid connectors, these drawbacks are outweighed by the longer life of the restoration.
